# RETRACTED: Sarfstein et al. Identification of Insulin-Like Growth Factor-I Receptor (IGF-IR) Gene Promoter-Binding Proteins in Estrogen Receptor (ER)-Positive and ER-Depleted Breast Cancer Cells. *Cancers* 2010, *2*, 233–261

**DOI:** 10.3390/cancers17213567

**Published:** 2025-11-04

**Authors:** Rive Sarfstein, Antonino Belfiore, Haim Werner

**Affiliations:** 1 Department of Human Molecular Genetics and Biochemistry, Sackler School of Medicine, Tel Aviv University, Tel Aviv 69978, Israel; 2 Department of Clinical and Experimental Medicine, University Magna Graecia of Catanzaro, 88100 Catanzaro, Italy

The journal retracts the article “*Identification of Insulin-Like Growth Factor-I Receptor (IGF-IR) Gene Promoter-Binding Proteins in Estrogen Receptor (ER)-Positive and ER-Depleted Breast Cancer Cells*” [1] cited above.

Following publication, concerns were brought to the attention of the Editorial Office regarding the integrity of the data presented in this publication [1].

Adhering to our standard procedure, an investigation was conducted by the Editorial Office and Editorial Board that identified indications of inappropriate image editing and partial duplication in Figures 3, 5 and 6 of this article. While the authors collaborated within this process, raw material meeting the journals requirements for original images https://www.mdpi.com/journal/cancers/instructions#oriimages could not be provided for Editorial Board Evaluation. Consequently, the Editorial Board has lost confidence in the reliability of the findings and has decided to retract this publication, as per MDPI’s retraction policy (https://www.mdpi.com/ethics#_bookmark30).

This retraction was approved by the Editor-in-Chief of the journal Cancers.

The authors did not agree to this retraction.
